# “Thumbnail Patch” Endoaneurysmorrhaphy Technique: Essentials for Modified Circular Repair of Left Ventricular Aneurysm

**DOI:** 10.1016/j.atssr.2025.11.019

**Published:** 2025-12-15

**Authors:** Kerem M. Vural, Safak Alpat

**Affiliations:** Department of Cardiovascular Surgery, Hacettepe University Hospitals, Ankara, Turkey

## Abstract

Among the various repair methods proposed for left ventricular aneurysms, those that use a patch sutured along a circular line are the most popular. Many variants have been developed, but our “thumbnail” circular patch endoaneurysmorrhaphy technique incorporates refinements for optimum restoration of the distorted geometry and size, including the use of a significantly smaller patch, strict adherence to the fibrotic transitional border regardless of its depth, and deliberate spatial arrangement of the patch. These refinements minimize stress on the suture line and optimize ventricular remodeling. This paper provides a comprehensive visual and textual explanation of these essential surgical steps.

Left ventricular aneurysms (LVAs), mechanical complications of acute myocardial infarction with serious hemodynamic, arrhythmic, and thromboembolic consequences, are rarely seen today thanks to the widespread availability and liberal use of primary percutaneous coronary intervention techniques. The rarity of this severe condition means that opportunities to observe its surgical management remain limited for both surgical residents and experienced surgeons, thus underscoring the importance of detailed case reports.

The surgical repair of LVAs includes plication, resection with linear closure, septoplasty, and circular patch endoaneurysmorrhaphy (CPE) techniques.^1^ Many variations of patch-employing circular repair techniques have been proposed since the 1980s, developed around the same basic principles.^2^^,^^3^ Our “thumbnail” CPE technique, however, incorporates refinements that are essential for excellent spherical restoration of the distorted geometry and size, even in very large anterior LVAs (Figure 1A). The “thumbnail patch” refers to a considerably smaller patch, deliberately sized to be slightly larger than a human thumbnail, designed to maximize hemodynamic benefits and prevent additional dyskinetic regions.

**Figure 1:**
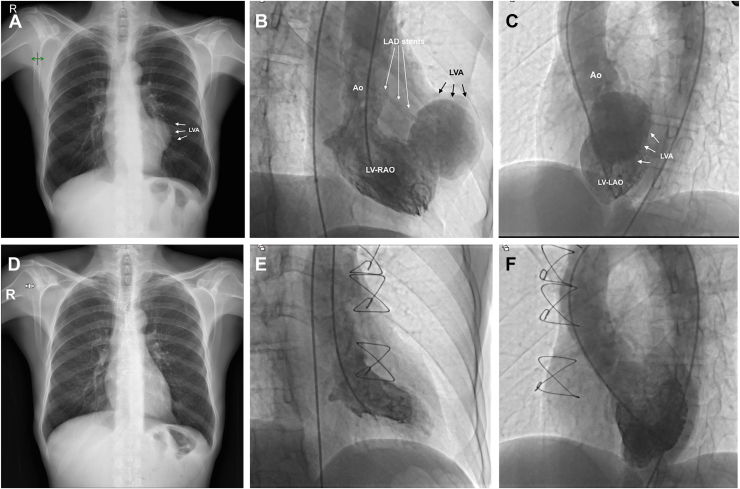
(A) Preoperative chest roentgenogram; arrows depict a large left ventricular aneurysm (LVA). (B) Preoperative left ventriculography, right anterior oblique (RAO) view, systole. The in-line multiple stenting of the left anterior descending (LAD) artery in a local hospital during the patient’s initial emergency admission with persistent ventricular fibrillation and vigorous resuscitation gives a pipeline (full-metal jacket) appearance. (C) Preoperative left ventriculography, left anterior oblique (LAO), systole. (D) Postoperative chest roentgenogram. (E) Postoperative left ventriculography, right anterior oblique view, systole. (F) Postoperative left ventriculography, left anterior oblique view, systole. (Ao, aorta; LV, left ventricle; R, patient’s right.)

## Technique

Candidates for thumbnail CPE are best evaluated by biplane left ventriculography. A right anterior oblique projection demonstrates the segmentary contraction pattern and delineates the borders of the aneurysm (Figure 1B, Video 1). Whereas a right anterior oblique projection helps determine whether a CPE-type repair is *needed*, left anterior oblique views determine whether such a repair is *feasible*. The latter provides essential prognostic information because the left lateral wall contractions indicate the reserve pump function after aneurysmectomy (Figure 1C, Video 1). If this segment is poorly contracting, an unfavorable outcome may ensue.

The CPE technique is best performed using cardioplegic arrest with the patient undergoing moderately hypothermic cardiopulmonary bypass. Coronary artery bypass grafting may be added, usually performed before the aneurysm repair, either on the involved territory^1^ or on other sections that are at risk as a result of extensive coronary atherosclerosis.

**Figure 2:**
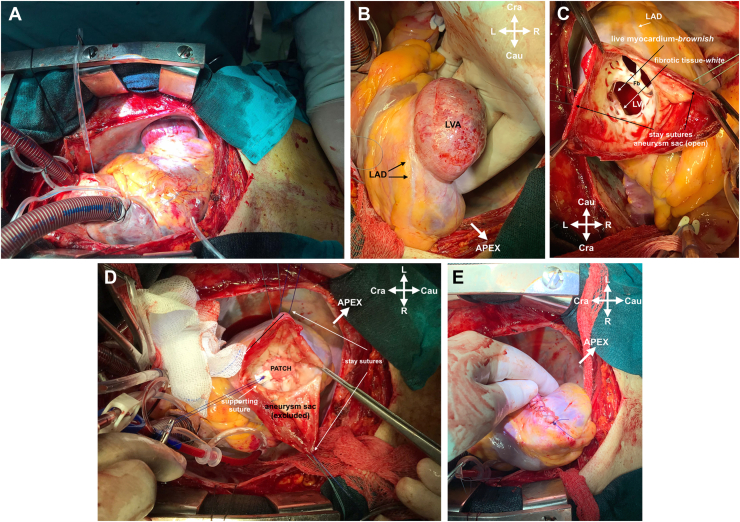
(A) Aneurysm adhered to the pericardium. (B) Pericardial adhesions are released. (C) Aneurysmal sac’s interior, exposed. (D) The patch is placed; the aneurysmal sac is exteriorized. (E) The ventriculotomy is closed over the patch, leaving minimal dead space. (Cau, caudal; Cra, cranial; Fb, thick fibrotic muscle band to be resected; L, patient’s left; LAD, left anterior descending (artery); LV, left ventricle; LVA, left ventricular aneurysm; R, patient’s right.)

Thin-walled aneurysms often adhere firmly to the pericardium; therefore, pericardiotomy should be done with care, slightly rightward, without attempting to release these adhesions before aortic cross-clamping, to avoid catastrophic bleeding and minimize the risk of air embolism with an open left ventricle (Figure 2A). The dyskinetic movements come into view (Video 2, part 1). This segment collapses with unloading the heart on cardiopulmonary bypass. The pericardial adhesions are released, and the aneurysm is fully exposed (Figure 2B).

The sac is opened through a 5- to 6-cm longitudinal apical ventriculotomy between 2 parallel stay sutures. Fibrotic bands are liberally resected until fully exposing the ventricular cavity. The scarred tissue border lying deep on the endocardium is identified. This is almost always not a zone but more like a line, sharply delineating the transition between the white fibrotic tissue and the brownish live myocardium (Figure 2C). The patch will be anchored along this line.

One notable aspect of our technique is the use of a considerably smaller patch, even for aneurysms with very large necks. A tear-shaped patch, 2.5 × 2 cm, is cut from Teflon felt or similar material. This patch should be kept very small, slightly larger than a thumbnail (hence the name “thumbnail”) to maximize hemodynamic benefits. By doing so, we prevent the creation of another large, dyskinetic region while maintaining the “circularity” of the closure line and the spherical shape of the final product. Hence, a purposeful purse-string effect is obtained without transforming the closure line into a linear one. Other techniques use either unnecessarilly large patches or no patch at all (ie, directly closing the edge with a purse-string), both causing too much stress on the closure line (Laplace's Law). A smaller patch, by decreasing the overall radius of curvature, actually *reduces* the wall tension according to Laplace’s law (the tension on the wall of a sphere is directly proportional to the radius). The purse-string effect helps achieve this desired smaller radius, thereby actually reducing tension on the suture line, in contrast to larger patches or linear closures, which create higher stress.

The patch is anchored with a continuous 2-0 polypropylene suture, double-armed with round-bodied, sharply curved, small needles, proceeding circumferentially along the fibrotic border with sparsely placed, semitangential, chunky bites. We prefer to use a thick, monofilament, continuous suture for more uniform distribution of contractile stress along the suture line, rather than using “separate” buttressed sutures, which are preferred in other techniques.

Another noteworthy difference of our technique is the strict adherence to the fibrotic transitional border, regardless of how deeply it delves into the ventricle. This suture line may appear quite deep on the septal side, which may raise concerns for excessively reducing the ventricular cavity. Nevertheless, it is essential to follow the fibrotic border closely, regardless of its depth. No diastolic volume measurements or estimates are necessary at this point. Eliminating all dyskinetic segments is essential because a well-repaired ventricle will perform surprisingly well, even if its volume is significantly reduced. The inefficient, akinetic aneurysmal segment impairs forward flow by stealing a significant stroke volume portion. Attempts to create more volume often leave behind a noncontractile, dead space, which not only fails to improve function but also places additional strain on the ventricle. By eliminating this nonfunctional dead space and restoring efficient geometry, the heart can pump more effectively, resulting in an increase in “*forward*” stroke volume, even if the total end-diastolic volume appears reduced compared with the diseased state, which included the nonfunctional aneurysm.

The spatial arrangement of the patch is also important. Larger patches used in other techniques sit in a more or less frontal plane, with the lateral edge angled toward the back. However, because the scar on the septum is deeper than on the lateral wall, and our suture precisely follows the scar line, our patch is positioned rather obliquely, almost in a “sagittal” orientation, with the medial (septal) edge tilted backward and the lateral edge pointing forward (Figure 2D). It is a natural or geometric result of anchoring the patch deeper on the septum than on the lateral wall. The sagittal orientation confirms that the deeper infarct border on the septum is strictly followed. Therefore, the choice of sagittal orientation is not arbitrary but is dictated by the anatomy of the scar and is essential for achieving optimal physiologic reconstruction and function, thus contrasting it with the limitations of a frontal orientation, which may not conform as well to the natural geometry and could lead to suboptimal outcomes.

The 2 suture arms tied over a supporting Teflon pledget, after eliminating any slack with a nerve hook. The heart is deaired by passing a large-bore needle through the patch (neoapex) while gently squeezing the heart in hand. For hemostatic purposes, the exteriorized fibrotic edges are brought together with a continuous suture, overlapping like the buttons on a double-breasted jacket (Figure 2E), thereby ensuring that there is no potential space for a hematoma. The aortic cross-clamp is then released. The patient is rewarmed, weaned from bypass, and decannulated in the usual manner (Video 2, part 2).

## Comment

Patients who require left ventricular aneurysmectomy constitute a high-risk group, and the guidelines for selecting those patients who are most likely to benefit from the repair are not well defined. Both early and late prognoses depend on the residual myocardium’s reserve function. Regardless of the technique used, the repair should aim to restore stroke volume, reverse the remodeling, eliminate the risk of rupture, and prevent further expansion, which can lead to mitral regurgitation secondary to papillary muscle displacement.

Applying linear closure techniques often results in a geometrically distorted left ventricle with an echocardiographically or angiographically evident akinetic segment along the repair line. Although circular repair techniques are generally more anatomically suitable, trimming the patch too large can also result in a noncontracting, dyskinetic segment.

The present technique offers a comprehensive repair that achieves excellent restoration of the distorted geometry and size (Figures 1D, 1E, 1F, Video 1) into a spherical, all-contracting chamber without leaving any residual dead space. The hospital mortality was 3.9% in a large series of ours, with 92% survival at 6 years and notable long-term symptomatic improvement.^1^
